# Supplementary material for: The C terminus of the mycobacterium ESX-1 secretion system substrate ESAT-6 is required for phagosomal membrane damage and virulence
Source: Proc Natl Acad Sci U S A. 2022 Mar 10;119(11):e2122161119. doi: 10.1073/pnas.2122161119 (PMC8931374; doi:10.1073/pnas.2122161119)
Supplement: Supplementary File [file pnas.2122161119.sapp.pdf]

Supplementary Information for:

The C terminus of the mycobacterium ESX-1 secretion system substrate ESAT-6 is required for phagosomal membrane damage and virulence

Morwan M. Osman, Jonathan K. Shanahan, Frances Chu, Kevin Takaki, Malte L. Pinckert, Antonio Pagán, Roland Brosch, William H. Conrad, Lalita Ramakrishnan

This PDF file includes:
Supplementary Figures 1 to 3
Supplementary Tables 1 to 3
Supplementary References

Supplementary Figures

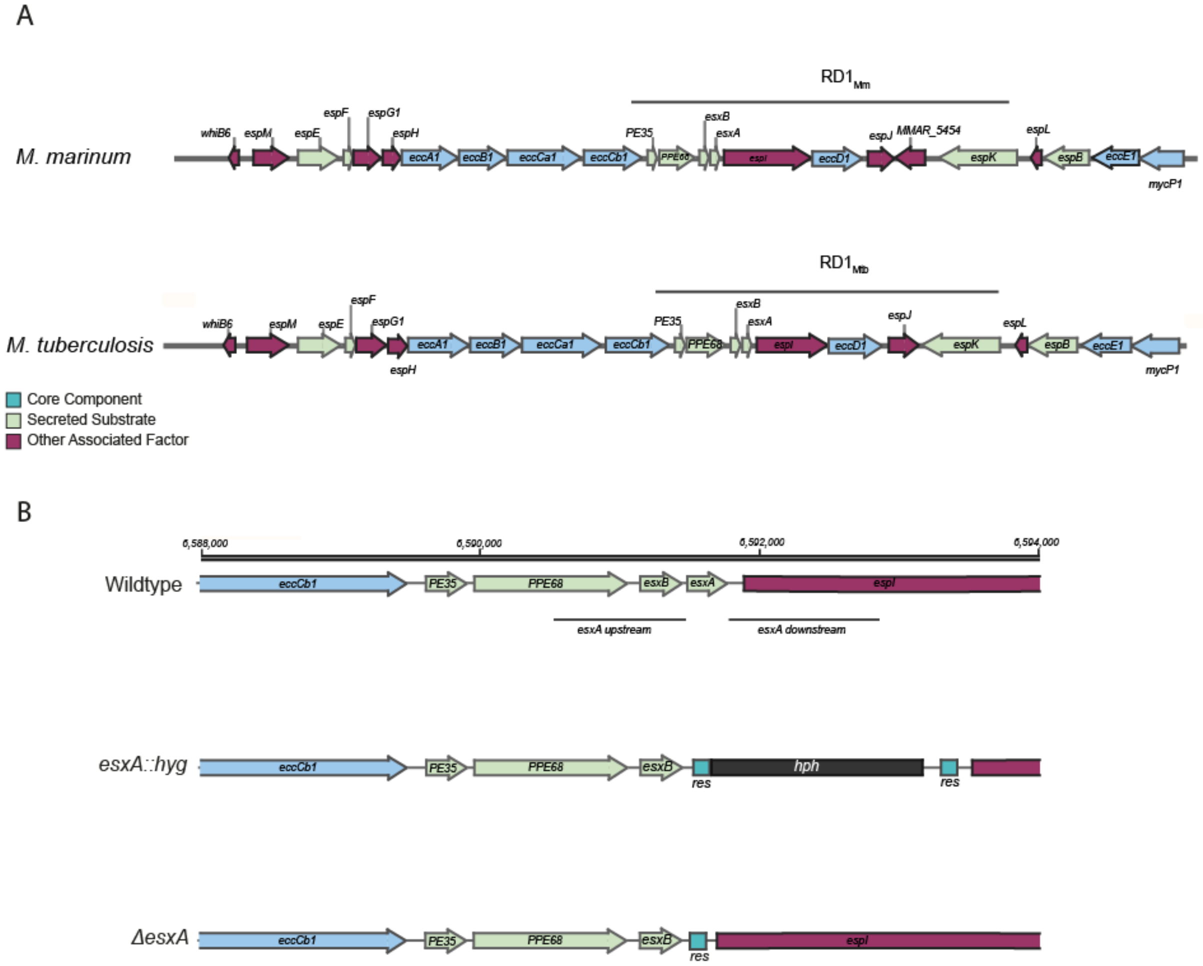

Figure S1. ESX-1 loci and scheme for Δ*esxA* mutant generation.
(**A**) Alignment of Mm and Mtb ESX-1 loci, with regions corresponding to RD1 deletions. (**B**) Schematic showing the initial, intermediate, and final alleles in the generation of the *esxA* mutant in Mm. (Top) Wildtype *esxA* loci with flanking region upstream and downstream *esxA* as targeted by the deletion construct. (Middle) Phage transduction was used to generate the *esxA::hyg* mutant with *esxA* replaced by the *res*-flanked *hph* gene encoding the hygromycin-B-phosphotransferase selectable marker. (Bottom) The *hph* gene was then excised by a gamma-delta resolvase, generating the unmarked Mm-*ΔesxA* mutant. Full details of the primers, plasmids and phasmids can be found in the Materials & Methods.

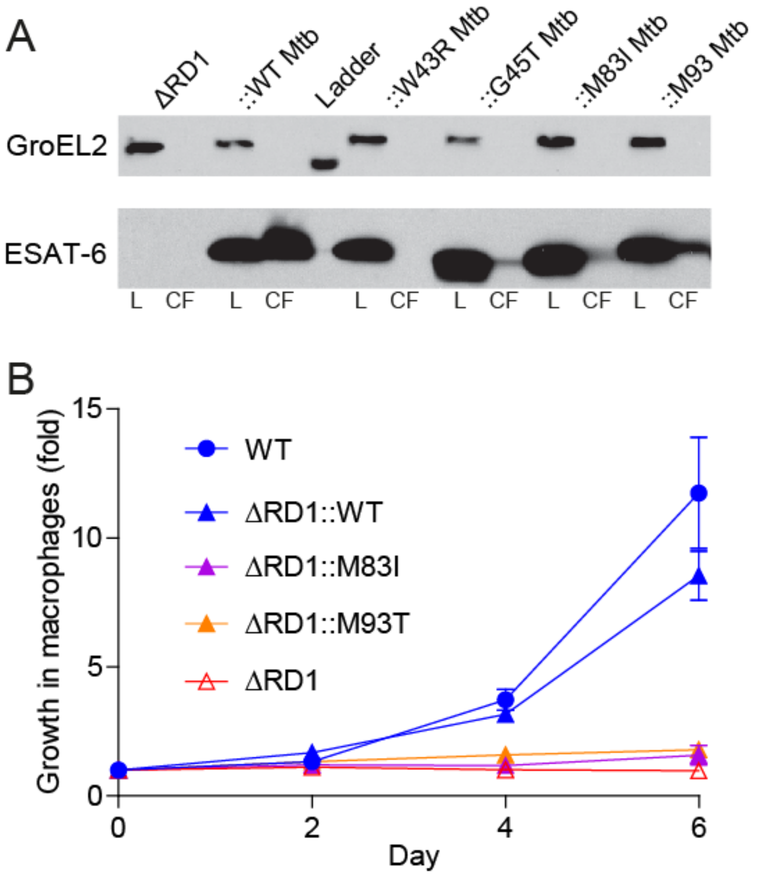

Figure S2. Mm ΔRD1::M83I$_{Mt}$ and ::M93T$_{Mt}$ mutants have reduced ESAT-6 secretion and fail to grow in macrophages.
(**A**) Immunoblot of Mm lysates (L) and culture filtrates (CF) at 48 hours. Data representative of three independent experiments. (**B**) Intramacrophage growth of Mm within J774A.1 cells as measured by bacterial fluorescence. Data representative of three independent experiments.

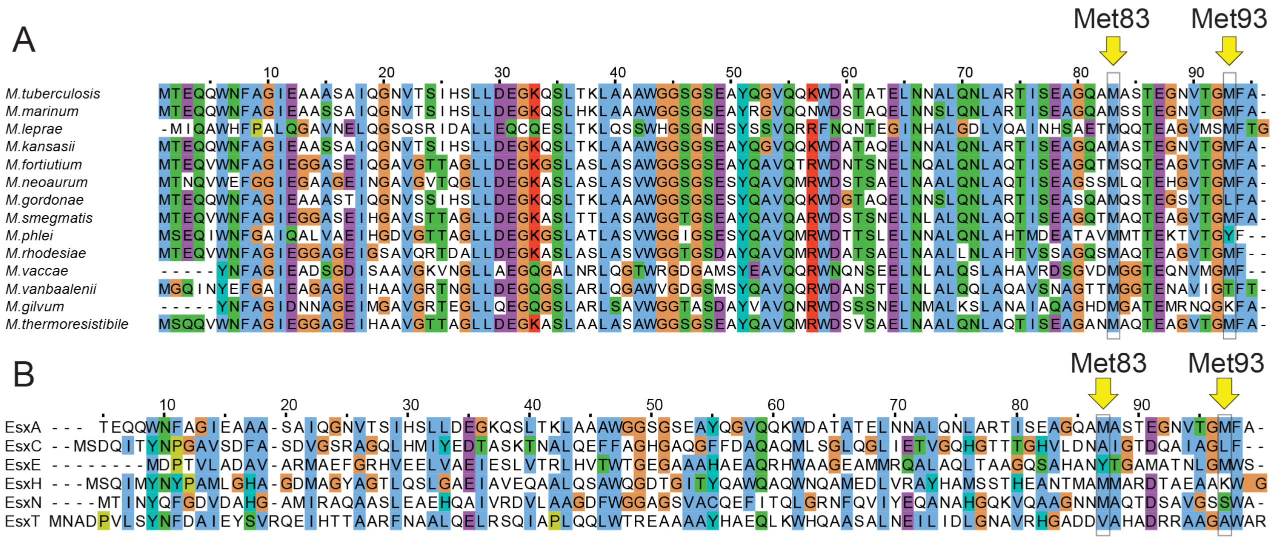

Figure S3. The C-terminal Met83 and Met93 residues of ESAT-6 are highly conserved. (**A**, **B**) Sequence alignment of Mtb ESAT-6 homologs (**A**) and paralogs (**B**). Yellow arrows denote residues aligned with Mtb ESAT-6 methionine 83 and 93.

Supplementary Tables

Table S1. Plasmids used in this study.

Number	Plasmid	Use	Resistance	Source
**1**	pTEC27	Mycobacterial plasmid containing the gene for the fluorescent protein tdTomato under the constitutive mycobacterial promoter msp12.	Hygromycin	Addgene #30182
**2**	pTEC31	pTEC27 with the hygromycin resistance marker replaced with kanamycin.	Kanamycin	(1)
**3**	pTEC35	Mycobacterial plasmid expressing the fluorescence protein EBFP2 under the msp12 promoter.	Kanamycin	(1)
**4**	pMH406	Complementation construct containing *M. tuberculosis esxBA* operon under control of the mycobacterial optimal promoter	Kanamycin	(2)
**5**	pMH406-M83I	pMH406 with a point mutation resulting in the expression of ESAT-6 M83I	Kanamycin	This study
**6**	pMH406-M93T	pMH406 with a point mutation resulting in the expression of ESAT-6 M93T	Kanamycin	This study
**7**	ESAT-6-BEI	Purification of his-tagged EsxA	Ampicillin	BEI
**8**	CFP10-BEI	Purification of his-tagged EsxB	Ampicillin	BEI
**9**	pFC52	Expression of his-tagged EsxA mutant M93T.	Kanamycin	This study
**10**	phAE159	Shuttle phasmid for phage production.	Ampicillin	(3)
**11**	pYUB854	Contains a hygromycin cassette flanked by the γδ-resolvase sites.	Hygromycin	(3)
**12**	pYUB854-*esxA*	Plasmid 11 containing sequences upstream and downstream sequences flanking esxA.	Hygromycin	This study
**13**	phSP105	Plasmid 10 with PacI fragments from plasmid 12 subcloned in.	Hygromycin	This study
**14**	pYUB870	Plasmid containing the γδ-resolvase gene (*tnpR*) from transposon Tn1000 under the control of the mycobacterial *hsp60* promoter.	Kanamycin	(3)
**15**	2F9-*esxA*-WT	2F9 integrating cosmid containing the Mtb ESX-1 locus (bp 4,336,809-4,368,613).		(4)
**16**	2F9-*esxA*-W43R	Plasmid 15 with a mutation in *esxA* resulting in the production of ESAT-6-W43R.		(4)
**17**	2F9-*esxA*-G45T	Plasmid 15 with a mutation in *esxA* resulting in the production of ESAT-6-G45T		(4)
**18**	2F9-*esxA*-M83I	Plasmid 15 with a mutation in *esxA* resulting in the production of ESAT-6-M83I		(4)
**19**	2F9-*esxA*-M93T	Plasmid 15 with a mutation in *esxA* resulting in the production of ESAT-6-M93T		(4)

Table S2. Primers used in this study.

Primer Name	Sequence	Purpose
**-955esxA_LF_SpeI_F**	GCCACTAGTGTCACAGGTCACCGGCATAC	Cloning of upstream sequence of esxA for phage transduction.
**-23esxA_LF_XhoI_R**	GTTCCTCGAGCGTTTTAGGGGAATCAGAAGC	Cloning of upstream sequence of esxA for phage transduction.
**+301esxA_RF_AgeI_F**	AGGCACCGGTTTCGCGTAGAATACCGAAGC	Cloning of downstream sequence of esxA for phage transduction.
**+1191esxA_RF_XbaI_R**	GCCTCTAGAGGAGCCGGTGGCAGTT	Cloning of downstream sequence of esxA for phage transduction
**esxA_SBprobe_F**	GCATACCGAGCAGTGAGCTT	Southern blot probe for confirming insertion of hyg cassette into esxA
**esxA_SBprobe_R**	GCCAAATTGTTGGCAAGTCT	Southern blot probe for confirming insertion of hyg cassette into esxA
**esxBA_Mm_Junct_F**	gaggcaggtaatttcgagcg	PCR of Mm genomic region containing the junction between esxB and esxA
**esxBA_Mm_Junct_R**	ggtttgcccagtttcgtcat	PCR of Mm genomic region containing the junction between esxB and esxA
**WC047**	CTATGCGAACGTCCCAGTGAC	Site-directed mutagenesis of esxA to generate M93T
**WC048**	GTCACTGGGACGTTCGCATAG	Site-directed mutagenesis of esxA to generate M93T mutant
**WC067**	CTTCGGTCGAAGCTATTGCCTGACCG	Site-directed mutagenesis of esxA to generate M83I mutant
**WC068**	CGGTCAGGCAATAGCTTCGACCGAAG	Site-directed mutagenesis of esxA to generate M83I mutant
**K2A**	GGCCAGCGAGCTAACGAGACNNNNGTTGC	Arbitrary primer
**K2B**	GGCCAGCGAGCTAACGAGACNNNNGATAT	Arbitrary primer
**K2C**	GGCCAGCGAGCTAACGAGACNNNNAGTAC	Arbitrary primer
**TnMarR3**	ACAACAAAGCTCTCACCAACCGTG	Corresponds to one end of the TnMarMme transposon
**K3**	GGCCAGCGAGCTAACGAGAC	Fixed; corresponds to the set 5' end of K2 primers
**TnMarR2**	CAGACACTGCTTGTCCGATATTTGATTTAGG	Corresponds to one end of the TnMarMme transposon (nested, internal to product produced by TnMarR3)

Table S3. Mycobacterial strains used in this study.

	Strain	Description	Resistance	Source
**1**	M strain	Wildtype M. marinum	None	ATCC
**2**	mc^{2}6206	*M. tuberculosis* H37Rv *ΔpanCD ΔleuCD,*	None	(5)
**3**	Δ*esxA*	M strain with a deletion of the *esxA* gene.	None	This study
**4**	*eccA1*::Tn	Transposon mutant 19729 containing transposon disrupting the *eccA1* gene	Hygromycin	This study
**5**	Δ*esxA::esxA*$_{Mtb}$	Strain 3 complemented with plasmid 4.	Kanamcyin	This study
**6**	Δ*esxA::esxA*-M83I$_{Mtb}$	Strain 3 complem::M93T$_{Mtb}$ ented with plasmid 5.	Kanamcyin	This study
**7**	Δ*esxA*::*esxA*-M93T$_{Mtb}$	Strain 3 complemented with plasmid 6. Also called ::M93T$_{Mtb}$	Kanamcyin	This study
**8**	Δ*esxA* + pTEC27	Strain 3 with plasmid 1.	Hygromycin	This study
**9**	Δ*esxA::esxA* + pTEC27	Strain 5 with plasmid 1	Hygromycin Kanamycin	This study
**10**	Δ*esxA::esxA*-M83I$_{Mtb}$ + pTEC27	Strain 6 with plasmid 1.	Hygromycin Kanamycin	This study
**11**	Δ*esxA*::*esxA*-M93T$_{Mtb}$ + pTEC27	Strain 7 with plasmid 1.	Hygromycin Kanamycin	This study
**12**	*eccA1*::Tn + pTEC31	Strain 4 with plasmid 2.	Hygromycin Kanamycin	This study
**13**	M strain + pTEC35	Wildtype M. marinum transformed with plasmid 3.	Kanamycin	This study
**14**	mc^{2}6206 + pTEC35	mc^{2}6206 transformed with plasmid 3.	Kanamycin	This study

References:

1. K. Takaki, J. M. Davis, K. Winglee, L. Ramakrishnan, Evaluation of the pathogenesis and treatment of Mycobacterium marinum infection in zebrafish. *Nat Protoc* **8**, 1114-1124 (2013).
2. K. M. Guinn *et al.*, Individual RD1-region genes are required for export of ESAT-6/CFP-10 and for virulence of Mycobacterium tuberculosis. *Mol Microbiol* **51**, 359-370 (2004).
3. S. Bardarov *et al.*, Specialized transduction: an efficient method for generating marked and unmarked targeted gene disruptions in Mycobacterium tuberculosis, M. bovis BCG and M. smegmatis. *Microbiology (Reading)* **148**, 3007-3017 (2002).
4. P. Brodin *et al.*, Functional analysis of early secreted antigenic target-6, the dominant T-cell antigen of Mycobacterium tuberculosis, reveals key residues involved in secretion, complex formation, virulence, and immunogenicity. *J Biol Chem* **280**, 33953-33959 (2005).
5. C. Vilcheze *et al.*, Rational Design of Biosafety Level 2-Approved, Multidrug-Resistant Strains of Mycobacterium tuberculosis through Nutrient Auxotrophy. *mBio* **9** (2018).
